# Quality‐of‐life comparison between intensity‐modulated proton therapy and volumetric‐modulated arc therapy in patients with nasopharyngeal carcinoma: Preliminary findings from real‐world data

**DOI:** 10.1002/cam4.7421

**Published:** 2024-06-22

**Authors:** Ching‐Nung Wu, Yu‐Ming Wang, Wei‐Chih Chen, Jung‐Der Wang, Shau‐Hsuan Li, Chung‐Feng Hwang, Yun‐Hsuan Lin, Sheng‐Dean Luo, Chung‐Ying Lin

**Affiliations:** ^1^ Department of Otolaryngology Kaohsiung Chang Gung Memorial Hospital and Chang Gung University College of Medicine Kaohsiung Taiwan; ^2^ Department of Public Health, College of Medicine National Cheng Kung University Tainan Taiwan; ^3^ Department of Radiation Oncology Kaohsiung Chang Gung Memorial Hospital and Chang Gung University College of Medicine Kaohsiung Taiwan; ^4^ Proton and Radiation Therapy Center Kaohsiung Chang Gung Memorial Hospital and Chang Gung University College of Medicine Kaohsiung Taiwan; ^5^ Department of Occupational and Environmental Medicine, College of Medicine National Cheng Kung University Tainan Taiwan; ^6^ Department of Hematology‐Oncology Kaohsiung Chang Gung Memorial Hospital and Chang Gung University College of Medicine Kaohsiung Taiwan; ^7^ Graduate Institute of Clinical Medical Sciences, College of Medicine Chang Gung University Taoyuan Taiwan; ^8^ School of Traditional Chinese Medicine Chang Gung University College of Medicine Taoyuan Taiwan; ^9^ Institute of Allied Health Sciences, College of Medicine National Cheng Kung University Tainan Taiwan; ^10^ Department of Occupational Therapy, College of Medicine National Cheng Kung University Tainan Taiwan; ^11^ Biostatistics Consulting Center, National Cheng Kung University Hospital, College of Medicine National Cheng Kung University Tainan Taiwan

**Keywords:** EAT‐10, nasopharyngeal carcinoma, proton beam therapy, quality of life, radiotherapy

## Abstract

**Background:**

Limited data are available to examine the effects of intensity‐modulated proton therapy (IMPT) on patient‐reported outcomes in patients with nasopharyngeal carcinoma (NPC). Thus, we assessed whether patients receiving IMPT reported a better short‐term quality of life (QoL) than those receiving volumetric‐modulated arc therapy (VMAT).

**Methods:**

We consecutively invited newly diagnosed NPC patients who had undergone standard treatment protocol within 2 years post‐radiotherapy at our institute between 2021 and 2023 to participate in the observational study. All participants completed the EuroQol five‐dimension, World Health Organization Quality‐of‐Life—Brief, Sinonasal Outcome Test 22, Eustachian Tube Dysfunction Questionnaire‐7, and Eating Assessment Tool‐10 (EAT‐10) questionnaires. QoL functions were estimated using a kernel‐smoothing method. A linear mixed model introducing the inverse probability of treatment weighting was constructed to estimate the effect of IMPT versus VMAT.

**Results:**

We identified 94 patients who completed 120 QoL assessments. Participants receiving IMPT were younger and had higher levels of education and higher household income. QoL functions showed that post‐treatment EAT‐10 scores consistently appeared significantly lower for IMPT than VMAT. After adjusting for factors including age, gender, education, household income, and cancer stages, patients receiving IMPT consistently showed significantly better QoL scores in EAT‐10, indicative of a medium effect. Additionally, factors such as age, household income level, and treatment regimen might influence either generic or condition‐specific QoL.

**Conclusions:**

Patients receiving IMPT demonstrated potentially improved eating‐related QoL compared to those receiving VMAT within a 2‐year post‐radiotherapy period. However, larger head‐to‐head comparison studies are warranted to confirm these findings.

## 1 INTRODUCTION

Nasopharyngeal carcinoma (NPC) is a relatively rare form of cancer but has a higher incidence in specific regions like Southeast Asia and Southern China.^1^ The primary treatment for NPC is radiotherapy (RT), often used in conjunction with chemotherapy.^2^ However, the anatomical closeness of the nasopharynx to the cranial base presents challenges in RT administration. This spatial closeness results in a range of locoregional complications, such as chronic rhinosinusitis (CRS), auditory impairment, eustachian tube dysfunction (ETD), and deglutition difficulties, thereby substantially impacting the overall quality of life (QoL) for affected patients.^3^ Currently, intensity‐modulated proton therapy (IMPT), an advanced form of proton beam RT, has demonstrated potential in reducing treatment‐related toxic effects in NPC patients.^4^, ^5^

Proton beam RT is characterized by its capacity to administer a minimal radiation dosage to the surrounding healthy tissues, while concentrating the bulk of its energy within the designated tumor region. It facilitates superior dose decay and substantially safeguards healthy tissue.^6^ In contrast, traditional photon‐based RT continues to expose a considerable radiation dose along its trajectory.^7^ This unique characteristic of proton beam RT presents a distinct advantage when treating NPC due to the tumor's close proximity to complex anatomical structures. Indeed, IMPT has been shown to reduce acute grade 2 or higher adverse events by 50% to 80% compared with volumetric‐modulated arc therapy (VMAT), a leading photon RT method, in NPC patients.^4^, ^5^ However, the patient‐reported outcome (PRO), particularly in this novel domain of using IMPT for NPC treatment, remains a crucial missing piece in comprehensive healthcare assessment. As a result, the incorporation of PRO has become a top priority to provide a more comprehensive evaluation of patient toxic effects.^8^

The primary objective of this observational study was to assess whether patients receiving IMPT exhibit superior QoL (including generic and condition‐specific QoL) compared to those undergoing VMAT from real‐world data. Additionally, we sought to identify any other patient characteristics that might predict a better QoL following RT.

## 2 MATERIALS AND METHODS

### 2.1 Patient recruitment and data collection

This research, being an ongoing project, endeavor received formal authorization from the Ethical Review Committee of Kaohsiung Chang Gung Memorial Hospital (KCGMH), as evidenced by the reference numbers 201901691B0C601 and 202200543B0C502. Prior to the study's commencement, documented informed consent was duly secured from each individual electing to participate in the study. Patients with newly diagnosed NPC, who had undergone standard protocol RT within 2 years at KCGMH between April 2021 and September 2023, were consecutively invited to participate. Among them, those who were found with distant metastasis in the initial pre‐treatment cancer status were excluded. Additionally, patients who suffered from major comorbidities that might significantly impair QoL, such as end‐stage kidney disease (ESRD), hemiplegia or paraplegia, decompensated stage of liver cirrhosis, and another terminal‐stage cancer, were also excluded. Each participant was not restricted to a single survey; however, each was conducted at least one month apart. During the survey, patients' demographics, socioeconomic status, and clinical disease status were also collected.

### 2.2 Standard treatment protocol and radiation modalities – VMAT or IMPT

In adherence to the guidelines set forth by the National Comprehensive Cancer Network (NCCN) for NPC, patients diagnosed at stage I, as per the American Joint Committee on Cancer's (AJCC) eighth edition, were subjected to RT as a monotherapeutic approach. Conversely, patients categorized within stages II to IVA underwent a regimen of concurrent chemoradiotherapy. The radiation dosages administered to the gross tumor volume, anatomical sites deemed high‐risk, and those considered low‐risk were 69.96, 56 to 59.4, and 52.8 Gray equivalent (GyE), respectively.^9^ These dosages were dispensed over the course of 33 daily fractions. Concurrent chemotherapy involved weekly cisplatin (40 mg/m^2^) for up to 7 cycles. Induction chemotherapy was chosen for patients with stages II‐IVa NPC, in accordance with the NCCN Guidelines.

IMPT plans were generated using the RayStation (RaySearch Laboratories, Stockholm, Sweden) with the pencil beam line scanning system. Most patients received treatment with three beams and robust optimization, accounting for 3 mm setup uncertainties and 3.5% range uncertainties. The clinical target volumes (CTVs) were delineated based on our institutional protocol and the treatment objectives aimed to cover 99.5% of the clinical target volumes with the prescribed dose while minimizing radiation doses to adjacent organs at risk.^10^ For VMAT, plans were generated utilizing at least dual complete arcs. The planning target volume (PTV) margin of 3 mm was incorporated into each of the corresponding CTVs to account for potential uncertainties. The optimization criteria ensured that 95% of the PTV were encompassed by the prescribed dose, which was set at 100% of the target dose.^10^ In Taiwan, NPC treatment with VMAT is covered by national health insurance (NHI), resulting in minimal out‐of‐pocket expenses. However, IMPT is not covered by NHI, and its usage to treat NPC incurs full out‐of‐pocket expenses. Radiation oncologists would thoroughly explain the advantages and disadvantages of the two approaches to patients before initiating RT, allowing patients to freely choose between IMPT or VMAT.

### 2.3 QOL questionnaires

#### 2.3.1 Generic QOL

Two questionnaires were used to measure generic QoL: The European Quality of Life‐5 Dimensions (EQ‐5D) and the World Health Organization Quality of Life – BREF (WHOQOL‐BREF). The EQ‐5D questionnaire serves as a preference‐oriented tool designed for the quantification of QoL utility indices, systematically assessing five distinct dimensions: mobility, self‐care, usual activities, pain/discomfort, and anxiety/depression, each with three severity levels.^11^ In the context of Taiwan's evaluative framework, these health state metrics are converted into a utility index that spans a continuum from 0 to 1. In this scale, a score of 0 is indicative of mortality, while a score of 1 signifies optimal health.^12^

The WHOQOL‐BREF questionnaire,^13^ a generic psychometric instrument with demonstrated sensitivity to head and neck cancer patients, was also employed with good psychometric properties.^14^ It assigns scores from 1 to 5 for each facet, with higher scores indicating better QoL. The questionnaire delineates overall QoL and general health, as well as specific domains: physical, psychological, social relationships, and environment. Domain scores were calculated by multiplying the average of all facets in the same domain by four, ranging from 4 to 20.

#### 2.3.2 Condition‐specific QOL

For evaluating otorhinolaryngologic‐related QoL, three questionnaires were employed: the SNOT‐22, Eustachian Tube Dysfunction Questionnaire‐7 (ETDQ‐7), and Eating Assessment Tool‐10. The SNOT‐22, a comprehensive instrument encompassing 22 evaluative components, functioned as a metric for assessing therapeutic outcomes in subjects afflicted with chronic sinonasal disorders.^15^ Wu et al.^16^ proposed a framework comprising five domains—nasal, ear/facial, sleep, functional, and emotional—for the evaluation of otorhinologic‐associated QoL in individuals diagnosed with NPC utilizing the SNOT‐22. Each item on the SNOT‐22 was scored from 0 to 5, with higher scores indicating either deteriorated patient functionality or aggravated symptom severity. The cumulative score spanned a continuum from 0 to 110.^15^

The ETDQ‐7 was utilized to evaluate ETD in adult subjects—a condition recognized to be concomitant with otologic and rhinologic manifestations subsequent to RT targeting the nasopharyngeal area.^17^, ^18^ Each query within the ETDQ‐7 was evaluated using a seven‐tiered Likert scale, where a score of 1 denoted an absence of issues, and a score of 7 represented the most severe symptomatic experience. The total scores on the ETDQ‐7 reflected ETD severity, ranging from 7 to 49.

Assessing dysphagia‐specific QoL, the Eating Assessment Tool‐10 (EAT‐10) was self‐administered by patients.^19^ As subjective and objective swallowing abnormalities are common after RT for NPC,^20^ the EAT‐10 effectively assessed dysphagia. It comprised 10 items scored from 0 (no impairment) to 4 (severe impairment), with total scores ranging from 0 to 40; higher scores indicated greater self‐perceived impairment.^19^

### 2.4 QoL function after RT

For each evaluation of QoL, the temporal interval post‐RT was delineated as the duration extending from the initiation of RT to the date of the QoL assessment. To model the temporal trajectory of QoL, Gaussian kernel‐smoothing techniques were employed.^21^ Specifically, at any given temporal juncture denoted as *t*, the mean QoL estimate for that moment was ascertained through a weighted mean of QoL assessments, where the weights were governed by a parameter termed as bandwidth. In this study, the bandwidth was configured at 0.1. Bootstrap resampling was used to establish the pertinent confidence intervals for the mean QoL function estimations. For each specific time point, a 95% confidence interval was constructed based on the 2.5th and 97.5th percentiles derived from 1000 bootstrapped mean QoL estimates.

### 2.5 Covariates

The survey recorded various demographic and clinical variables, including age at diagnosis, gender, education, marital status, family monthly income, comorbidities using the Charlson Comorbidity Index (CCI), and Karnofsky Performance Status Scale. Information on disease status, including clinical staging according to the AJCC 8th staging system (TNM staging system), treatment regimen (chemotherapy utilization), and recurrence status, was collected from chart review. As the study aimed to explore the association between different RT modalities and post‐RT QoL, confounders—variables affecting both the use of RT modalities and QoL—included age at RT, gender, education, family monthly income, and disease severity (e.g., AJCC staging). Covariates such as recurrence status and performance status at the time of QoL assessment were considered intermediate variables between RT and QoL, rather than confounders (Figure S1).

### 2.6 Statistical analysis

Between‐group differences (IMPT vs. VMAT) were evaluated utilizing the *χ*_2_ test or Fisher exact test for categorical covariates and Student's *t*‐test or Mann–Whitney *U*‐test for parametric or nonparametric continuous variables. Linear mixed models, with subject and time after RT as random factors, were employed to investigate the determinants of QoL for repeated assessments within individual subjects. Confounders were included in constructing the multivariable regression models. In the evaluation of generic QoL, the EQ‐5D and domains within the WHOQOL‐BREF were treated as dependent variables. Within these models, positive coefficients indicated a variable's prediction of better QoL metrics. Conversely, for condition‐specific QoL—using total and domain scores of the SNOT‐22, scores of the ETDQ‐7, and EAT‐10 as dependent variables—a positive coefficient signified a variable's prediction of worsened QoL metrics. To assess clinical significance, effect sizes were calculated using Cohen's d due to the absence of established minimal clinically important differences for the instruments in question among NPC patients.^22^

To strengthen the robustness of our findings beyond mere adjustment through multivariable regression, we utilized the inverse probability of treatment weighting (IPTW) method to address the observed confounding between the two groups.^23^ Initially, we calculated the probability of receiving the intervention (IMPT vs. VMAT) based on individual characteristics, including age, gender, education level, household income, marital status, belief, CCI, AJCC 8th stage, and treatment regimen (i.e., propensity score). Subsequently, we calculated the weights as the inverse of the propensity score and applied them in the linear mixed model to further balance the two groups. The statistical analyses were performed using R version 4.2.1 and the Statistical Analysis System® software version 9.4 (SAS Institute, Cary, NC, USA). All reported *p*‐values were two‐sided.

## 3 RESULTS

Initially, one hundred and two patients were approached for potential inclusion in the study. After excluding four individuals who opted out, two patients with initial distant metastasis, and two patients with major comorbidities (one with hemiplegia and another with a different terminal cancer), 94 participants who received either VMAT or IMPT as primary treatment participated in the study. All 94 patients completed at least one instance of all the questionnaires. Some patients repeatedly completed the QoL questionnaires, resulting in a total of 120 QoL assessments for data analysis. Table 1 delineates the characteristics of the study participants, categorized by the therapeutic interventions they received. Patients who received IMPT were younger and had a higher proportion of individuals with advanced education and greater household income. The severity of disease status also varied between the two groups. The distribution frequency of QoL assessments post‐RT initiation was similar between the two groups, ranging from 3 to 24 months.

**TABLE 1:** Baseline characteristics of patients stratified by radiation modality.

Participant characteristics	IMPT (*n* = 46)	VMAT (*n* = 48)	*p* value^a^
Age at RT; median (IQR); years	45.5 (41.0–56.0)	51.5 (42.5–61.5)	0.06
Gender; *n* (%)			
Female	13 (28.3)	8 (16.7)	0.17
Male	33 (71.7)	40 (83.3)	
Education level; *n* (%)			
High school diploma or less	14 (30.4)	35 (72.9)	<0.01
College degree or higher	32 (69.6)	13 (27.1)	
Marital status; *n* (%)			
Single	11 (23.9)	8 (16.7)	0.67
Married	31 (67.4)	35 (72.9)	
Divorced/separated/widowed	4 (8.7)	5 (10.4)	
Monthly household income ($); *n* (%)			
<2000	12 (26.1)	24 (50.0)	<0.01
2000–4000	19 (41.3)	22 (45.8)	
>4000	15 (32.6)	2 (4.2)	
Charlson comorbidity index; *n* (%)			
0–1	35 (76.1)	31 (64.6)	0.22
≥2	11 (23.9)	17 (35.4)	
Tumor status; *n* (%)			
T0	0 (0.0)	1 (2.1)	0.34
T1	25 (54.4)	22 (45.8)	
T2	4 (8.7)	6 (12.5)	
T3	11 (23.9)	7 (14.6)	
T4	6 (13.0)	12 (25.0)	
Nodal status; *n* (%)			
N0	8 (17.4)	7 (14.6)	0.01
N1	15 (32.6)	21 (43.8)	
N2	21 (45.6)	10 (20.8)	
N3	2 (4.4)	10 (15.8)	
Clinical AJCC staging; *n* (%)			
I	6 (13.0)	5 (10.4)	<0.01
II	10 (21.8)	13 (27.1)	
III	22 (47.8)	9 (18.8)	
IVa	8 (17.4)	21 (43.7)	
Treatment regimen; *n* (%)			
RT alone	6 (13.0)	5 (10.4)	0.69
Induction chemotherapy plus CRT	40 (87.0)	43 (89.6)	
Questionnaire acquiring			
Number; *n* (%)	61 (50.8)	59 (49.2)	
Time since the start of radiotherapy; median (IQR); months	12.0 (10.0–18.0)	14.0 (10.0–19.0)	0.25

Abbreviations: AJCC, American Joint Committee on Cancer; CRT, chemoradiotherapy; IMPT, intensity‐modulated proton therapy; IQR, interquartile range; RT, radiotherapy; VMAT, volumetric‐modulated arc therapy.

^a^ Categorical variables were assessed by *χ*_2_ test or Fisher exact test. Parametric or nonparametric continuous variables were compared by Student's *t*‐test or Mann–Whitney *U*‐test, respectively.

Figure S2 depicts the temporal variations in utility values, and generic QoL scores across the four domains within WHOQOL‐BREF between IMPT and VMAT over a two‐year post‐RT period. Compared to VMAT, IMPT's utility values exhibited a trend toward being significantly higher starting around 18 months post‐ RT initiation, although the difference narrowly missed statistical significance due to overlapping confidence intervals (i.e., shaded areas). Using linear mixed models, we examined the overall impact of IMPT versus VMAT on generic QoL (Table 2). Both crude and adjusted analyses indicated that patients receiving IMPT had higher utility values, though these differences were not statistically significant. No significant QoL score differences were observed across all domains in the WHOQOL‐BREF between the two treatments.

**TABLE 2:** Regression coefficients for generic quality of life derived from mixed model analyses of the EQ‐5D and WHOQOL‐BREF.

	IMPT versus VMAT		
	Crude	Adjustment^a^	IPTW adjustment^b^
EQ‐5D: Utility value	0.031 (0.016)/0.07	0.035 (0.020)/0.09	0.035 (0.019)/0.07
WHOQOL‐ BREF: total score	−0.01 (0.45)/0.97	0.57 (0.55)/0.31	0.62 (0.52)/0.24
Overall quality of life	−0.07 (0.15)/0.61	−0.10 (0.20)/0.62	0.04 (0.18)/0.63
General health	0.13 (0.18)/0.47	0.32 (0.22)/0.17	0.34 (0.21)/0.11
Physical domain	0.19 (0.53)/0.70	0.62 (0.65)/0.35	0.68 (0.62)/0.28
Psychological domain	−0.27 (0.54)/0.61	0.31 (0.67)/0.64	0.27 (0.62)/0.66
Social domain	0.20 (0.53)/0.70	0.97 (0.62)/0.13	1.02 (0.59)/0.09
Environment domain	−0.18 (0.46)/0.68	0.38 (0.55)/0.50	0.49 (0.52)/0.36

*Note*: Values outside the parentheses represent regression coefficients, while those inside the parentheses denote standard errors. The number following the slash is the *p*‐value.

Abbreviations: EQ‐5D, European Quality of Life‐5 Dimensions; IMPT, intensity‐modulated proton therapy; IPTW, inverse probability of treatment weighting; VMAT, volumetric‐modulated arc therapy; WHOQOL‐ BREF, World Health Organization Quality of Life‐BREF.

^a^ In the adjustment model, potential confounders including age, gender, educational level, family monthly income, and disease severity were controlled for.

^b^ In the IPTW‐adjusted model, treatment weights were applied, and the aforementioned confounders were duly controlled.

**FIGURE 1:**
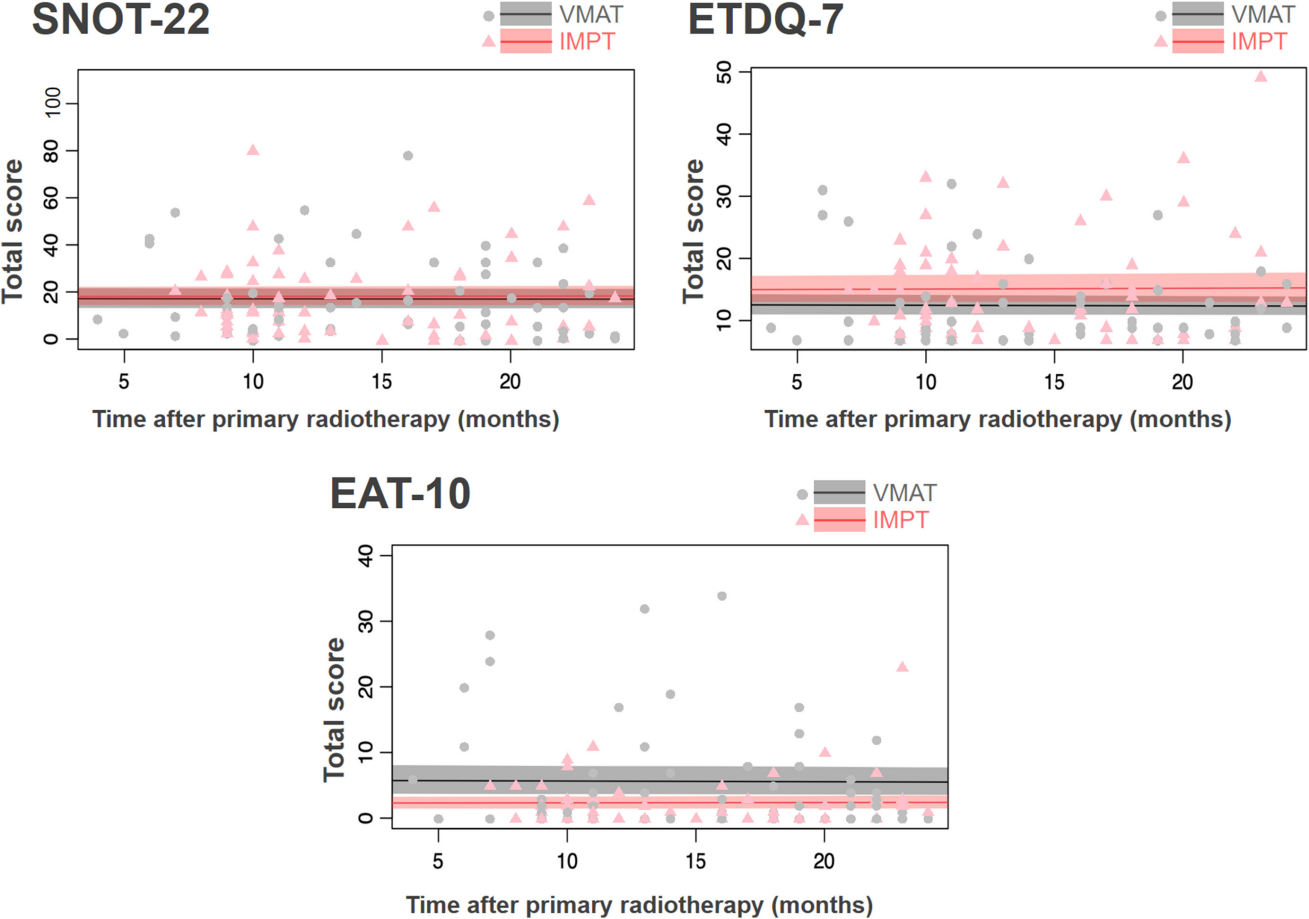
Condition‐specific QoL scores based on the SNOT‐22, ETDQ‐7, and EAT‐10 questionnaires within 2 years after the initiation of radiotherapy, comparing IMPT to VMAT. Each dot represents an individual survey. The colored shadow represents a 95% confidence interval for each QoL function. Overlap of the shaded areas during any time period indicates a lack of statistical significance between IMPT and VMAT for that interval; conversely, non‐overlapping shaded areas signify statistical significance. EAT‐10, Eating Assessment Tool‐10; ETDQ‐7, Eustachian Tube Dysfunction Questionnaire‐7; IMPT, intensity‐modulated proton therapy; QoL, quality of life; SNOT‐22, Sinonasal Outcome Test 22; VMAT, volumetric‐modulated arc therapy.

In terms of condition‐specific QoL scores, patients treated with IMPT consistently registered lower scores on the EAT‐10 throughout the two‐year post‐RT period, suggesting an improved QoL related to eating following RT (Figure 1). Regarding the comparative effects of IMPT versus VMAT on condition‐specific QoL (Table 3), no significant differences were observed in the SNOT‐22 and the ETDQ‐7 scores between the two patient groups. On the other hand, both the crude and adjusted linear mixed models showed statistically significant lower scores in EAT‐10. This indicates that patients receiving IMPT had a better QoL score related to eating after RT. Even after applying weighting to the regression model, the significant difference in EAT‐10 scores between IMPT and VMAT persisted (coef: −3.56, SE: 1.53, *p* = 0.03). The Cohen's d value was 0.49, indicating a medium effect.

**TABLE 3:** Regression coefficients for condition‐specific quality of life derived from mixed model analyses of the SNOT‐22, ETQD, and EAT‐10.

	IMPT versus VMAT		
	Crude	Adjustment^a^	IPTW adjustment^b^
SNOT‐22: total	−0.45 (3.45)/0.89	−3.22 (4.35)/0.46	−3.38 (4.24)/0.43
Nasal	0.18 (1.32)/0.88	−1.18 (1.65)/0.48	−1.09 (1.63)/0.51
Ear/facial	0.71 (0.65)/0.28	0.03 (0.81)/0.96	−0.03 (0.75)/0.96
Sleep‐related	−0.77 (1.04)/0.46	−0.75 (1.30)/0.57	−0.84 (1.27)/0.51
Functional	−0.46 (0.67)/0.50	−0.64 (0.86)/0.46	−0.69 (0.80)/0.39
Emotional	−0.06 (0.48)/0.89	−0.47 (0.61)/0.44	−0.48 (0.59)/0.42
ETDQ‐7	2.20 (1.56)/0.17	1.56 (1.99)/0.44	1.39 (1.87)/0.46
EAT‐10	−3.54 (1.31)/0.01*	−3.47 (1.61)/0.04*	−3.56 (1.53)/0.03*

*Note*: Values outside the parentheses represent regression coefficients, while those inside the parentheses denote standard errors. The number following the slash is the *p*‐value.

Abbreviations: EAT‐10, Eating Assessment Tool‐10; ETDQ‐7, Eustachian Tube Dysfunction Questionnaire‐7; IMPT, intensity‐modulated proton therapy; IPTW, inverse probability of treatment weighting; SNOT‐22, Sinonasal Outcome Test 22; VMAT, volumetric‐modulated arc therapy.

^a^ In the adjustment model, potential confounders including age, gender, educational level, family monthly income, and disease severity were controlled for.

^b^ In the IPTW‐adjusted model, treatment weights were applied, and the aforementioned confounders were duly controlled.

* *p* ≤ 0.05.

Regarding other factors associated with QoL post‐RT (Table 4), after adjusting for potential confounders, we found that with every 10‐year increase in age, the elderly population appeared to have better generic QoL scores in the social (coef: 0.70, SE: 0.25, *p* = 0.01), and environmental domains (coef: 0.54, SE: 0.22, *p* = 0.02). However, the elderly population was also found to have a poorer QoL score related to eating (coef: 1.33, SE: 0.65, *p* = 0.05). Additionally, patients treated with concurrent chemoradiotherapy (i.e., those who are not stage I) exhibited impairment in the general health facet of WHOQOL‐BREF (coef: −0.61, SE: 0.28, *p* = 0.04), but other domains of QoL were not affected. Lastly, higher socioeconomic status was not associated with better QoL, except that participants with higher income seemed to bear a better eating‐related QoL (coef: −2.65, SE: 1.30, *p* = 0.05).

**TABLE 4:** Regression coefficients for quality of life derived from mixed model analyses based on clinical characteristics.

	Age (every 10 years)	Gender (male vs. female)	Education (higher vs. lower)	Income (>2000 vs. <2000 $)	Treatment regimen (CRT vs. RT alone)
EQ‐5D: Utility value	−0.015 (0.008)	0.034 (0.020)	−0.002 (0.020)	0.004 (0.015)	−0.005 (0.025)
WHOQOL‐ BREF: total score	0.42 (0.22)	0.21 (0.53)	−0.89 (0.54)	0.72 (0.44)	0.12 (0.69)
Overall quality of life	−0.01 (0.07)	0.10 (0.19)	−0.21 (0.19)	0.25 (0.15)	−0.21 (0.19)
General health	−0.09 (0.09)	0.41 (0.22)	−0.33 (0.22)	0.26 (0.18)	−0.61 (0.28)*
Physical domain	−0.02 (0.26)	0.68 (0.64)	−1.00 (0.65)	0.81 (0.48)	−0.52 (0.82)
Psychological domain	0.46 (0.26)	0.27 (0.65)	−0.93 (0.66)	0.46 (0.55)	−0.37 (0.84)
Social domain	0.70 (0.25)*	−0.01 (0.61)	−0.71 (0.62)	0.69 (0.51)	1.39 (0.79)
Environment domain	0.54 (0.22)*	−0.10 (0.53)	−1.00 (0.54)	0.83 (0.45)	0.04 (0.69)
SNOT‐22: total	−0.12 (1.74)	−1.58 (4.30)	5.52 (4.33)	−4.15 (3.16)	−0.76 (5.48)
Nasal	0.23 (0.66)	−0.02 (1.62)	3.57 (1.63)*	−0.99 (1.24)	−0.17 (2.07)
Ear/facial	−0.35 (0.33)	−0.44 (0.81)	0.36 (0.82)	−0.36 (0.65)	−0.26 (1.04)
Sleep‐related	0.17 (0.53)	−0.78 (1.31)	1.05 (1.31)	−1.30 (0.88)	−1.18 (1.66)
Functional	−0.16 (0.34)	0.24 (0.84)	0.29 (0.85)	−1.17 (0.67)	0.75 (1.07)
Emotional	−0.05 (0.24)	−0.46 (0.60)	0.27 (0.60)	−0.36 (0.46)	0.13 (0.76)
ETDQ‐7	−0.92 (0.79)	0.81 (1.94)	0.85 (1.96)	−1.20 (1.50)	0.40 (2.48)
EAT‐10	1.33 (0.65)*	0.19 (1.59)	0.64 (1.62)	−2.65 (1.30)*	−0.36 (2.05)

*Note*: Values in parentheses are standard errors; The number following the slash indicates the *p*‐value.

Abbreviations: CRT, chemoradiotherapy; EAT‐10, Eating Assessment Tool‐10; EQ‐5D, European Quality of Life‐5 Dimensions; ETDQ‐7, Eustachian Tube Dysfunction Questionnaire‐7; RT, radiotherapy; SNOT‐22, Sinonasal Outcome Test 22; WHOQOL‐ BREF, World Health Organization Quality of Life‐BREF.

* *p* ≤ 0.05.

## 4 DISCUSSION

To the best of our knowledge, this study is among the first to evaluate QoL in patients receiving IMPT compared to those undergoing VMAT for NPC treatment. Our findings demonstrated that patients treated with IMPT had significantly lower scores on the EAT‐10, indicating a better QoL related to eating post‐RT. However, no significant differences were observed in WHOQOL‐BREF scores or other otorhinolaryngologic‐related QoL measures. Additionally, factors such as age, income level, and treatment regimen might influence either generic or condition‐specific QoL.

The preference for proton therapy in terms of PRO and QoL measures is well‐documented in the literature.^24^ These findings align with studies that have used provider‐rated toxicity measures.^25^ However, there remains a scarcity of studies concerning the PRO of proton therapy for NPC cases. In our study, patients receiving IMPT seemed to have a better utility value, but there was no significant difference in QoL scores compared to those receiving VMAT. Due to the absence of baseline data regarding pre‐treatment generic utility and QoL status in both the IMPT and VMAT groups, we cannot conclude that IMPT might offer improved utility or QoL compared to VMAT. Instead, these findings might stem from differences in background demographics. Socioeconomic status has been linked to subjective health and well‐being.^26^ However, Diwan et al. suggest that increasing material wealth beyond a certain level can negatively impact relational wealth.^27^ Given our findings that patients receiving IMPT were associated with higher income levels, the aforementioned literature might explain the relatively better utility, but not the improved QoL, in the IMPT group.

Our study also evaluated condition‐specific QoL tools to assess the facets that might benefit from IMPT. We found that patients receiving IMPT demonstrated persistently better eating‐related QoL within 2 years after RT, which can be attributed to the advantage of minimal dose delivery to normal tissues, such as salivary glands and larynx, by proton beam RT.^28^ Among our participants, six cases were assessed for dosimetric comparison between IMPT and VMAT in the oral cavity. The dose was nearly twice as high in the oral cavity for participants receiving VMAT, with the mean dose being 16.8 Gy in IMPT and 33.7 Gy in VMAT, respectively.^10^ Our findings were supported by previous literature, which showed that the mean dose to organs at risk between IMPT and photon‐based intensity‐modulated radiotherapy differed significantly in the tongue, mandible, and larynx, subsites related to food intake.^25^ Chou YC et al. also found that patients receiving IMPT are associated with reduced rates of nasogastric tube insertion and body weight loss, which could support our findings on eating‐related QoL.^5^ However, the nasal and ear/facial domains, which assessed post‐RT side effects of CRS and ETD, respectively, did not show significant differences between patients receiving IMPT and VMAT. The lack of significance might be attributed to the relatively short time interval between the questionnaire survey and the completion of RT, resulting in an insufficiently pronounced increase in post‐RT CRS and ETD proportions. In the existing literature, it has been demonstrated that a statistically significant rise is observed in SNOT‐22 scores, particularly in the nasal and ear/facial domains approximately 5 years after RT, indicating the potential development of bothersome otorhinologic‐related side effects in the late post‐RT phase.^16^, ^29^ Therefore, to validate the accuracy of the aforementioned conjecture, it is imperative to conduct further investigations comparing IMPT and VMAT over an extended duration.

Several limitations were present in our study. Firstly, the absence of pre‐treatment utility and QoL data for the IMPT and VMAT groups could affect the interpretation of our results. To mitigate this research gap, IPTW was employed in our models to equilibrate the characteristics of the two treatment groups (Table S2), thereby indirectly assuming comparable baseline characteristics, including pre‐treatment generic and condition‐specific QoL. Although this method does not ensure equivalency in baseline QoL measures, it represents our best effort to account for the missing baseline information. Secondly, concerns may arise regarding inferences drawn from a cross‐sectional study that incorporates different post‐RT time points, as well as repeated measurements within some individuals. To address this, we averaged the QoL function, as previous research has suggested that a random sample size of at least 50 is sufficient to estimate the mean QoL function in a cross‐sectional study.^30^ Furthermore, we employed a linear mixed model to account for the within‐subject correlation. Thirdly, the observational nature of our study design precludes definitive conclusions about causal relationships. Specifically, the lack of experimental manipulation means that not all potential biases, such as confounding and maturation effects, can be entirely ruled out. We attempted to adjust for all known confounders in the regression model, guided by the directed acyclic graph. (Figure S1) Additionally, our research serves as an exploratory study, offering compelling findings that merit further investigation and validation in future studies. Lastly, while our questionnaire utilized the SNOT‐22 and ETDQ‐7 to evaluate ear‐related symptoms, it did not address one of the significant impacts of NPC irradiation: early and late sensorineural hearing loss. In future research, a hearing‐specific questionnaire, such as the Hearing Handicap Inventory for Adults, might be considered for assessment.

## 5 CONCLUSION

In conclusion, our study revealed that patients receiving IMPT demonstrated significantly lower EAT‐10 scores, indicating improved eating‐related QoL within 2 years post‐RT. The findings highlight the potential benefit of IMPT over VMAT in treating NPC. However, considering the limitations of our study, further larger head‐to‐head comparison studies are necessary to validate and generalize these results.

## 6 DECLARATION OF GENERATIVE AI and AI‐ASSISTED TECHNOLOGIES IN THE WRITING PROCESS

During the preparation of this work the authors used ChatGPT4 in order to improve language and readability. After using this tool/service, the authors reviewed and edited the content as needed and takes full responsibility for the content of the publication.

## AUTHOR CONTRIBUTIONS

**Ching‐Nung Wu:** Conceptualization (lead); funding acquisition (lead); writing – original draft (lead). **Yu‐Ming Wang:** Investigation (lead). **Wei‐Chih Chen:** Visualization (lead). **Jung‐Der Wang:** Formal analysis (lead); software (lead). **Shau‐Hsuan Li:** Validation (lead). **Chung‐Feng Hwang:** Data curation (lead). **Yun‐Hsuan Lin:** Resources (lead). **Sheng‐Dean Luo:** Conceptualization (equal); project administration (lead); resources (equal). **Chung‐Ying Lin:** Methodology (lead); writing – review and editing (lead).

## Supporting information


Figure S1.

Figure S2.

Table S1.

Data S1.

## Data Availability

The data that support the findings of this study are available from the corresponding author upon reasonable request.

## References

[1] Chang ET , Ye W , Zeng Y‐X , Adami H‐O . The evolving epidemiology of nasopharyngeal carcinoma. Cancer Epidemiol Biomarkers Prev. 2021;30(6):1035‐1047. doi:10.1158/1055-9965.Epi-20-1702 33849968

[2] Chen YH , Luo SD , Wu SC , et al. Clinical characteristics and predictive outcomes of recurrent nasopharyngeal carcinoma—a lingering pitfall of the long latency. Cancer. 2022;14(15):3795. doi:10.3390/cancers14153795 PMC9367553 35954458

[3] McDowell L , Corry J , Ringash J , Rischin D . Quality of life, toxicity and unmet needs in nasopharyngeal cancer survivors. Front Oncol. 2020;10:930. doi:10.3389/fonc.2020.00930 32596155 PMC7303258

[4] Li X , Kitpanit S , Lee A , et al. Toxicity profiles and survival outcomes among patients with nonmetastatic nasopharyngeal carcinoma treated with intensity‐modulated proton therapy vs intensity‐modulated radiation therapy. JAMA Netw Open. 2021;4(6):e2113205. doi:10.1001/jamanetworkopen.2021.13205 34143193 PMC8214161

[5] Chou YC , Fan KH , Lin CY , et al. Intensity modulated proton beam therapy versus volumetric modulated arc therapy for patients with nasopharyngeal cancer: a propensity score‐matched study. Cancer. 2021;13(14):3555. doi:10.3390/cancers13143555 PMC8307135 34298769

[6] Patel SH , Wang Z , Wong WW , et al. Charged particle therapy versus photon therapy for paranasal sinus and nasal cavity malignant diseases: a systematic review and meta‐analysis. Lancet Oncol. 2014;15(9):1027‐1038. doi:10.1016/s1470-2045(14)70268-2 24980873

[7] Liu CM , Cheng JY , Lin YH , Chen CS , Wang YM . Elective upper‐neck versus whole‐neck irradiation of the uninvolved neck in patients with nasopharyngeal carcinoma. Lancet Oncol. 2022;23(6):e240. doi:10.1016/s1470-2045(22)00263-7 35654056

[8] Falchook A . Intensity‐modulated radiation therapy and intensity‐modulated proton therapy‐2 effective treatment modalities for nasopharyngeal cancer. JAMA Netw Open. 2021;4(6):e2113650. doi:10.1001/jamanetworkopen.2021.13650 34143197

[9] Taheri‐Kadkhoda Z , Björk‐Eriksson T , Nill S , et al. Intensity‐modulated radiotherapy of nasopharyngeal carcinoma: a comparative treatment planning study of photons and protons. Radiat Oncol. 2008;3(1):4. doi:10.1186/1748-717X-3-4 18218078 PMC2265732

[10] Lin YH , Cheng JY , Huang BS , et al. Significant reduction in vertebral artery dose by intensity modulated proton therapy: a pilot study for nasopharyngeal carcinoma. J Pers Med. 2021;11(8):822. doi:10.3390/jpm11080822 34442466 PMC8400425

[11] Szende A , Oppe M , Devlin N . EQ‐5D Value Sets: Inventory, Comparative Review and User Guide. Springer; 2007. 36810025

[12] Lee HY , Hung MC , Hu FC , Chang YY , Hsieh CL , Wang JD . Estimating quality weights for EQ‐5D (EuroQol‐5 dimensions) health states with the time trade‐off method in Taiwan. J Formos Med Assoc. 2013;112(11):699‐706. doi:10.1016/j.jfma.2012.12.015 24183199

[13] Yao G , Chung CW , Yu CF , Wang JD . Development and verification of validity and reliability of the WHOQOL‐BREF Taiwan version. J Formos Med Assoc. 2002;101(5):342‐351. 12101852

[14] Lin CY , Yang SC , Lai WW , Su WC , Wang JD . Rasch models suggested the satisfactory psychometric properties of the World Health Organization quality of life‐brief among lung cancer patients. J Health Psychol. 2017;22(4):397‐408. doi:10.1177/1359105315603474 26349615

[15] Hopkins C , Gillett S , Slack R , Lund VJ , Browne JP . Psychometric validity of the 22‐item Sinonasal Outcome Test. Clin Otolaryngol. 2009;34(5):447‐454. doi:10.1111/j.1749-4486.2009.01995.x 19793277

[16] Wu CN , Wang YM , Chen WC , et al. Evaluation of Sinonasal Outcome Test (SNOT‐22) domains in the assessment of the quality of life in patients with nasopharyngeal carcinoma. Cancer Manag Res. 2023;15:719‐728. doi:10.2147/cmar.S416353 37485039 PMC10362877

[17] Young YH , Hsieh T . Eustachian tube dysfunction in patients with nasopharyngeal carcinoma, pre‐ and post‐irradiation. Eur Arch Otorrinolaringol. 1992;249(4):206‐208. doi:10.1007/bf00178470 1642877

[18] McCoul ED , Anand VK , Christos PJ . Validating the clinical assessment of eustachian tube dysfunction: the Eustachian Tube Dysfunction Questionnaire (ETDQ‐7). Laryngoscope. 2012;122(5):1137‐1141. doi:10.1002/lary.23223 22374681 PMC3612400

[19] Belafsky PC , Mouadeb DA , Rees CJ , et al. Validity and reliability of the Eating Assessment Tool (EAT‐10). Ann Otol Rhinol Laryngol. 2008;117(12):919‐924. doi:10.1177/000348940811701210 19140539

[20] Hughes PJ , Scott PM , Kew J , et al. Dysphagia in treated nasopharyngeal cancer. Head Neck. 2000;22(4):393‐397. 10862024 10.1002/1097-0347(200007)22:4<393::aid-hed13>3.0.co;2-2

[21] Hwang JS , Wang JD . Integrating health profile with survival for quality of life assessment. Qual Life Res. 2004;13(1):1‐10; discussion 11‐4. doi:10.1023/b:Qure.0000015299.45623.38 15058782

[22] Cohen J . Statistical Power Analysis for the Behavioral Sciences. Lawrence Erlbaum Associates; 1988.

[23] Chesnaye NC , Stel VS , Tripepi G , et al. An introduction to inverse probability of treatment weighting in observational research. Clin Kidney J. 2021;15(1):14‐20. doi:10.1093/ckj/sfab158 35035932 PMC8757413

[24] Verma V , Simone CB 2nd , Mishra MV . Quality of life and patient‐reported outcomes following proton radiation therapy: a systematic review. J Natl Cancer Inst. 2018;110(4):341‐353. doi:10.1093/jnci/djx208 29028221

[25] Lewis GD , Holliday EB , Kocak‐Uzel E , et al. Intensity‐modulated proton therapy for nasopharyngeal carcinoma: decreased radiation dose to normal structures and encouraging clinical outcomes. Head Neck. 2016;38(Suppl 1):E1886‐E1895. doi:10.1002/hed.24341 26705956

[26] He Z , Cheng Z , Bishwajit G , Zou D . Wealth inequality as a predictor of subjective health, happiness and life satisfaction among Nepalese women. Int J Environ Res Public Health. 2018;15(12):2836. doi:10.3390/ijerph15122836 30545142 PMC6313399

[27] Diwan R . Relational wealth and the quality of life. Socioecon Rev. 2000;29(4):305‐340. doi:10.1016/S1053-5357(00)00073-1

[28] Kim JK , Leeman JE , Riaz N , McBride S , Tsai CJ , Lee NY . Proton therapy for head and neck cancer. Curr Treat Options Oncol. 2018;19(6):28. doi:10.1007/s11864-018-0546-9 29744681

[29] Hsin CH , Tseng HC , Lin HP , Chen TH . Post‐irradiation otitis media, rhinosinusitis, and their interrelationship in nasopharyngeal carcinoma patients treated by IMRT. Eur Arch Otorrinolaringol. 2016;273(2):471‐477. doi:10.1007/s00405-015-3518-8 25634060

[30] Hwang JS , Tsauo JY , Wang JD . Estimation of expected quality adjusted survival by cross‐sectional survey. Stat Med. 1996;15(1):93‐102. 8614748 10.1002/(SICI)1097-0258(19960115)15:1<93::AID-SIM155>3.0.CO;2-2

