# Supplementary figures and images for: Quality‐of‐life comparison between intensity‐modulated proton therapy and volumetric‐modulated arc therapy in patients with nasopharyngeal carcinoma: Preliminary findings from real‐world data
Source: Cancer Med. 2024 Jun 22;13(12):e7421. doi: 10.1002/cam4.7421 (PMC11192997; doi:10.1002/cam4.7421)

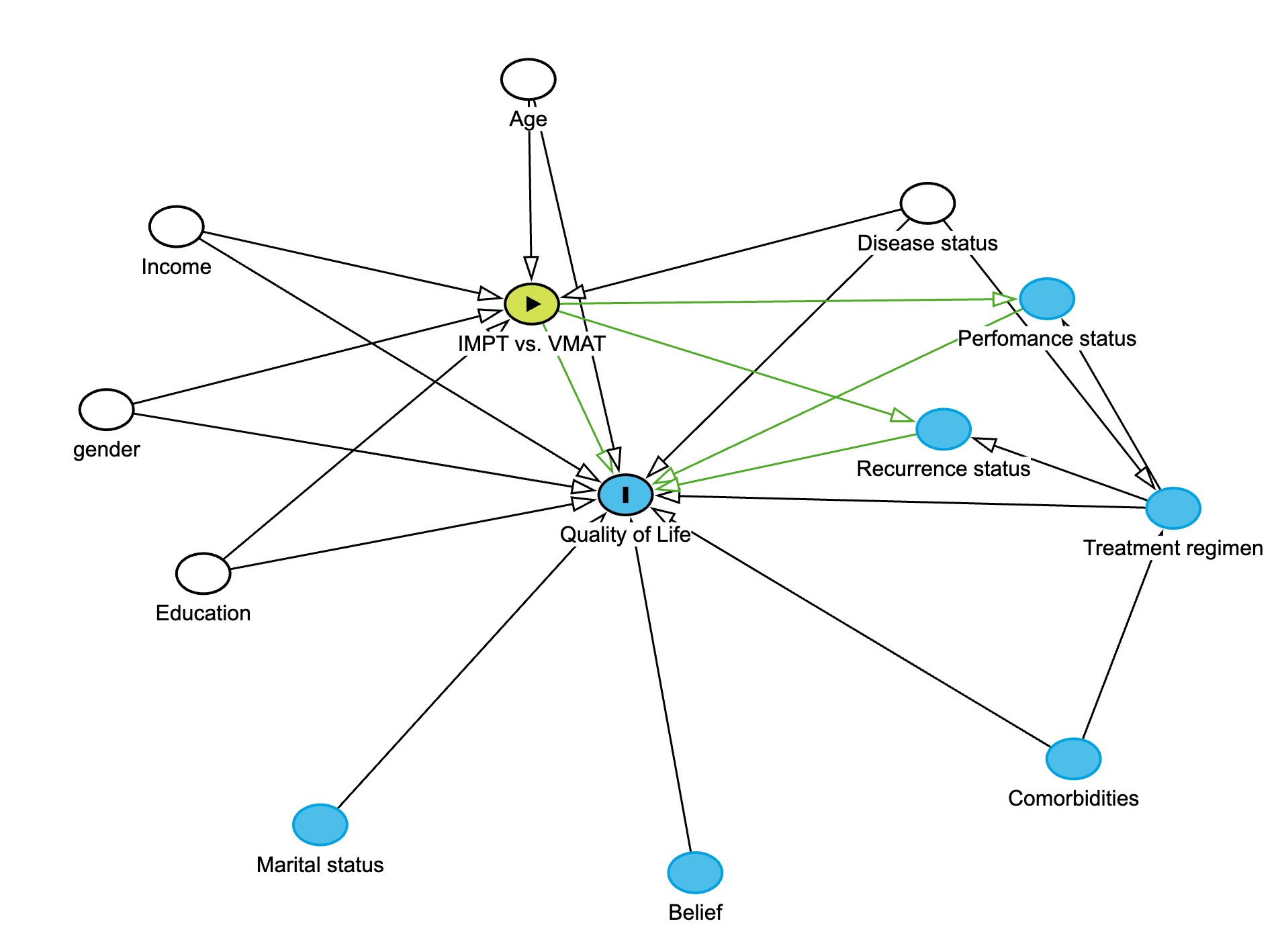

Supplement: Supplementary file 1 — Figure S1. [file CAM4-13-e7421-s001.tiff]

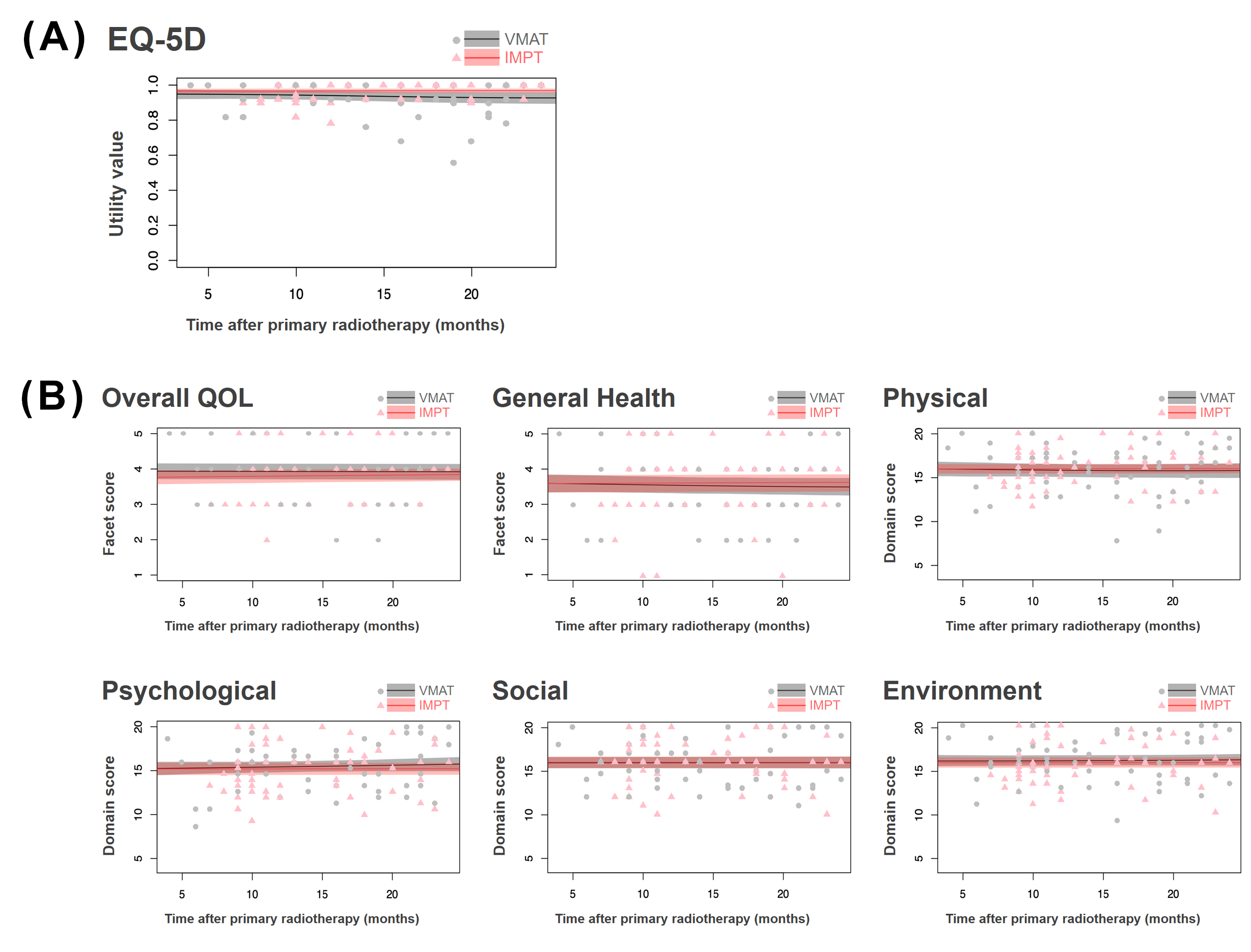

Supplement: Supplementary file 2 — Figure S2. [file CAM4-13-e7421-s004.tiff]
