# Supplementary material for: Quality‐of‐life comparison between intensity‐modulated proton therapy and volumetric‐modulated arc therapy in patients with nasopharyngeal carcinoma: Preliminary findings from real‐world data
Source: Cancer Med. 2024 Jun 22;13(12):e7421. doi: 10.1002/cam4.7421 (PMC11192997; doi:10.1002/cam4.7421)
Supplement: Supplementary file 3 — Table S1. [file CAM4-13-e7421-s003.docx]

**Table S1.** Values from all measured quality of life instruments, stratified by radiation modalities

|  | **IMPT** | | **VMAT** | |
| --- | --- | --- | --- | --- |
|  | Mean | Median (IQR) | Mean | Median (IQR) |
| **EQ-5D: Utility value** | 0.96 | 1.00 (0.91–1.00) | 0.93 | 1.00 (0.89–1.00) |
| **WHOQOL- BREF: total score** | 15.8 | 15.8 (13.9–17.9) | 15.9 | 15.9 (14.3–17.6) |
| Overall Quality of Life | 3.8 | 4.0 (3.0–4.0) | 3.9 | 4.0 (3.0–5.0) |
| General Health | 3.6 | 4.0 (3.0–4.0) | 3.5 | 4.0 (3.0–4.0) |
| Physical domain | 16.0 | 16.5 (14.2–17.1) | 15.8 | 16.5 (13.7–17.7) |
| Psychological domain | 15.2 | 16.0 (12.6–17.3) | 15.5 | 16.0 (13.3–17.3) |
| Social domain | 16.0 | 16.0 (14.6–18.0) | 15.9 | 16.0 (14.0–18.0) |
| Environment domain | 16.0 | 16.0 (14.6–17.7) | 16.2 | 16.0 (14.6–18.6) |
| **SNOT-22: total** | 18.2 | 12.0 (6.0–27.0) | 17.0 | 12.0 (4.0–24.0) |
| Nasal | 7.3 | 6.0 (2.0–11.0) | 6.2 | 5.0 (1.0–9.0) |
| Ear/facial | 2.7 | 2.0 (0.0–3.0) | 1.9 | 1.0 (0.0–3.0) |
| Sleep-related | 4.3 | 3.0 (0.0–8.0) | 4.9 | 3.0 (0.0–7.0) |
| Functional | 2.3 | 1.0 (0.0–3.0) | 2.6 | 1.0 (0.0–4.0) |
| Emotional | 1.3 | 0.0 (0.0–2.0) | 1.2 | 0.0 (0.0–1.0) |
| **ETDQ-7** | 15.1 | 12.0 (9.0–19.0) | 12.4 | 9.0 (8.0–15.0) |
| **EAT-10** | 2.3 | 1.0 (0.0–3.0) | 5.5 | 2.0 (0.0–7.0) |

Abbreviations: EAT-10, Eating Assessment Tool-10; EQ-5D, European Quality of Life-5 Dimensions; ETDQ-7, Eustachian Tube Dysfunction Questionnaire-7; IMPT, Intensity-modulated proton therapy; SNOT-22, Sinonasal Outcome Test 22; VMAT, volumetric-modulated arc therapy; WHOQOL- BREF, World Health Organization Quality of Life - BREF.

**Table S2.** Baseline characteristics of patients by radiation modality: pre- and post-IPTW adjustment

| **Characteristics** | **Before IPTW** | | | | **After IPTW** | | | |
| --- | --- | --- | --- | --- | --- | --- | --- | --- |
|  | VMAT | IMPT | Standardized  Mean  Difference | VMAT | | IMPT | Standardized  Mean  Difference |  |
| Age, mean (SD); years | 52.1 (12.2) | 47.6 (10.9) | 0.386 | 49.9 (11.6) | | 48.4 (10.3) | 0.138 |  |
| Gender;  Female  Male | 16.7%  83.3% | 28.3%  71.7% | 0.281 | 26.2%  73.8% | | 20.4%  79.6% | 0.137 |  |
| Education level (%)  High School Diploma or Less  College Degree or Higher | 72.9%  27.1% | 30.4%  69.6% | 0.939 | 60.4%  39.6% | | 51.0%  49.0% | 0.189 |  |
| Marital status (%)  Single  Married  Divorced / Separated / Widowed | 16.7%  72.9%  10.4% | 23.9%  67.4%  8.7% | 0.183 | 19.9%  70.9%  9.2% | | 25.2%  66.3%  8.6% | 0.127 |  |
| Monthly Household Income($) (%)  < 2000  2000-4000  > 4000 | 50.0%  45.8%  4.2% | 26.1%  41.3%  32.6% | 0.836 | 42.3%  42.4%  15.3% | | 42.0%  37.6%  20.5% | 0.144 |  |
| Charlson Comorbidity Index  0-1  ≥ 2 | 64.6%  35.4% | 76.1%  23.9% | 0.254 | 73.2%  26.8% | | 78.6%  21.4% | 0.125 |  |
| Tumor status (%)  T0  T1  T2  T3  T4 | 2.1%  45.8%  12.5%  14.6%  25.0% | 0%  54.3%  8.7%  23.9%  13.0% | 0.447 | 1.1%  52.2%  9.8%  16.7%  20.1% | | 0%  48.4%  8.0%  20.9%  22.8% | 0.207 |  |
| Nodal status (%)  N0  N1  N2  N3 | 14.6%  43.8%  20.8%  20.8% | 17.4%  32.6%  45.7%  4.3% | 0.702 | 12.3%  42.3%  28.0%  17.4% | | 14.3%  41.7%  34.7%  9.3% | 0.260 |  |
| Clinical AJCC staging (%)  I  II  III  IVa | 10.4%  27.1%  18.8%  43.8% | 13.0%  21.7%  47.8%  17.4% | 0.755 | 8.9%  31.2%  23.6%  36.3% | | 10.6%  25.9%  31.5%  32.1% | 0.203 |  |
| Treatment regimen; *n* (%)  RT alone  Chemoradiotherapy | 10.4%  89.6% | 13.0%  87.0% | 0.082 | 8.9%  91.1% | | 10.6%  89.4% | 0.055 |  |

Abbreviations: AJCC, American Joint Committee on Cancer; IMPT, Intensity-modulated proton therapy; RT, radiotherapy. VMAT, Volumetric Modulated Arc Therapy
