# Supplementary material for: Quality‐of‐life comparison between intensity‐modulated proton therapy and volumetric‐modulated arc therapy in patients with nasopharyngeal carcinoma: Preliminary findings from real‐world data
Source: Cancer Med. 2024 Jun 22;13(12):e7421. doi: 10.1002/cam4.7421 (PMC11192997; doi:10.1002/cam4.7421)
Supplement: Supplementary file 4 — Data S1. [file CAM4-13-e7421-s002.docx]

Figure S1 Directed acyclic graph of the associations between radiation modality and quality of life. IMPT, Intensity-modulated proton therapy; VMAT, volumetric-modulated arc therapy

Figure S2 Generic QoL scores within 2 years after the initiation of radiotherapy, comparing IMPT to VMAT. (A) EQ-5D (B) WHOQOL-BREF domains. Each dot represents an individual survey. The colored shadow represents a 95% confidence interval for each QoL function. Overlap of the shaded areas during any time period indicates a lack of statistical significance between IMPT and VMAT for that interval; conversely, non-overlapping shaded areas signify statistical significance.EQ-5D, European Quality of Life-5 Dimensions; IMPT, Intensity-modulated proton therapy; QoL, quality of life; VMAT, volumetric-modulated arc therapy; WHOQOL- BREF, World Health Organization Quality of Life-BREF
